# High-density genetic map and identification of QTLs for responses to temperature and salinity stresses in the model brown alga *Ectocarpus*

**DOI:** 10.1038/srep43241

**Published:** 2017-03-03

**Authors:** Komlan Avia, Susana M. Coelho, Gabriel J. Montecinos, Alexandre Cormier, Fiona Lerck, Stéphane Mauger, Sylvain Faugeron, Myriam Valero, J. Mark Cock, Pierre Boudry

**Affiliations:** 1 Algal Genetics Group, UMR 8227, CNRS, Sorbonne Universités, UPMC, Station Biologique Roscoff, CS 90074, 29688 Roscoff, France; 2 UMI 3614 Evolutionary Biology and Ecology of Algae, CNRS, Sorbonne Universités, UPMC, Pontificia Universidad Católica de Chile, Universidad Austral de Chile, Station Biologique Roscoff, CS 90074, 29688 Roscoff, France; 3 Centro de Conservación Marina and CeBiB, Facultad de Ciencias Biológicas, Pontificia Universidad Católica de Chile, Casilla 114-D, Santiago, Chile; 4 Ifremer, Laboratoire des Sciences de l’Environnement Marin (UMR 6539 LEMAR, UBO, CNRS, IRD, Ifremer), Centre Bretagne – ZI de la Pointe du Diable, CS 10070, 29280 Plouzané, France

## Abstract

Deciphering the genetic architecture of adaptation of brown algae to environmental stresses such as temperature and salinity is of evolutionary as well as of practical interest. The filamentous brown alga *Ectocarpus* sp. is a model for the brown algae and its genome has been sequenced. As sessile organisms, brown algae need to be capable of resisting the various abiotic stressors that act in the intertidal zone (e.g. osmotic pressure, temperature, salinity, UV radiation) and previous studies have shown that an important proportion of the expressed genes is regulated in response to hyposaline, hypersaline or oxidative stress conditions. Using the double digest RAD sequencing method, we constructed a dense genetic map with 3,588 SNP markers and identified 39 QTLs for growth-related traits and their plasticity under different temperature and salinity conditions (tolerance to high temperature and low salinity). GO enrichment tests within QTL intervals highlighted membrane transport processes such as ion transporters. Our study represents a significant step towards deciphering the genetic basis of adaptation of *Ectocarpus* sp. to stress conditions and provides a substantial resource to the increasing list of tools generated for the species.

Brown algae (Phaeophyceae) represent one of only five eukaryotic lineages that have evolved complex multicellularity independently. They are very distantly related to other major lineages such as animals or green plants^1^,^2^. These seaweeds constitute the dominant vegetation in the intertidal and subtidal zone of coastal ecosystems. They display a large variability of morphologies and some brown algae, such as kelps, may form extensive forests that offer suitable habitats for numerous species and hence support ocean biodiversity^3^,^4^. Brown algae have a wide range of uses as food, cosmetics or fertilizers and there is increasing attention on new biotechnological applications such as biofuels or cell-wall polysaccharides^5^,^6^,^7^. Despite their evolutionary, ecological and economic importance, many aspects of the biology of brown algae remain poorly understood. In particular, the range of phenotypic variation of such morphologically simple organisms, as well as the sources of this variation, their impact on fitness and the limits they may impose on adaptive responses to environmental conditions are largely unknown.

A large number of seaweed species are able to colonize the intertidal zone, and some of these species live only in this habitat. The intertidal zone is often characterized by the presence of multiple stresses including high temperature, low or high salinity, extreme irradiation with sunlight, wave mechanical force, desiccation and osmotic shocks due to tidal emersion-immersion cycles. There is evidence that the vertical distribution of organisms is related to their ability to tolerate different types of stress. This is particularly the case in the intertidal zone, where the difference between tolerant and susceptible species lies in the extent to which they can recover photosynthesis when re-immersed in seawater^8^. Coastal habitats may present other stresses, such as low salinity in estuarine environments, high concentrations of heavy metals and other chemical pollutants near ports, mines or coastal cities, spatial temporal variations in upwelling and other coastal oceanographic processes^9^,^10^. Adding to this complexity, variations in environmental conditions and their impact can even be observed at the microhabitat scale^11^. Remarkably, a number of seaweed species are able to cope with these strong environmental gradients. From an evolutionary point of view, two mechanisms can explain this tolerance to abiotic stressors: either evolution towards a generalist, all-purpose phenotype by increasing phenotypic plasticity, or local adaptation to specific sets of environmental conditions. It is however difficult to determine the relative importance of phenotypic plasticity and local adaptation, respectively, in determining differences among populations^12^. Plasticity may mimic local adaptation resulting in differences between populations that are not due to genetic factors^13^. While an extensive literature is available on the range of phenotypic plasticity in responses of seaweeds to both biotic and abiotic factors^14^, the genetic basis of their responses to environmental heterogeneity remains poorly explored. Several mechanisms involved in the responses to stressors such as heat, salinity and dehydration have been shown to be conserved among land plant taxa, including the presence of common actors such as reactive oxygen species (ROS), ion fluxes, activation of kinases and a cascade of reactions leading to the expression of transcription factors^15^,^16^,^17^. However, the extent to which mechanisms of stress tolerance known for those taxa are used by brown algae is largely unknown. Although some aspects might be conserved^18^,^19^, other important processes are likely to be found only in brown algae because of their unique evolutionary history. To understand the stress tolerance features characteristic of this group, it is therefore essential to study this phenomenon using brown algal models rather than extrapolating information from distant models in other phylogenetic groups. Such knowledge is of evolutionary as well as of applied interest, as novel biomolecules and metabolic pathways may be discovered. Furthermore, with the current expansion of the algal aquaculture sector, brown algae need to be well characterized for future domestication and selective breeding to provide the industry with optimized strains.

*Ectocarpus* sp., a small filamentous alga, has been established as the model organism for the brown algae, inducing the generation of several genetic and genomic resources such as a fully sequenced genome^1^, a genetic map^20^ and transcriptomic data^19^,^21^. Previously named *Ectocarpus siliculosus*, it has recently become clear that this taxa corresponds to a separate species that has not been described previously^22^ and we therefore refer to the species provisionally as *Ectocarpus* sp. 1c^23^, simplified as *Ectocarpus* sp. in the text below. As with many other members of the brown algal group, this species alternates between a haploid (gametophyte) and a diploid (sporophyte) phase, and thus its life cycle involves sequential development of two successive complex organisms^24^. A recent study in the *Ectocarpus* group has shown that haploid and diploid organisms also exhibit clear niche partitioning. However, mainly asexual populations also exhibit similar habitat usage^25^. Although brown algae are mainly marine species, rare cases of adaptation to fresh water have been reported^26^. An *Ectocarpus* species, *Ectocarpus subulatus*, is able to live in both marine and freshwater, suggesting extreme physiological plasticity^23^,^27^,^28^,^29^. Freshwater colonization is a rare event that is thought to induce rapid evolutionary radiations^30^. The colonization event involving this brown alga is still under investigation but it does not appear to have involved extensive radiation, as only about 1% of these species have colonized freshwater^31^. Recent analyses showed that the transition to low salinity has been accompanied by fundamental morphological, transcriptomic and metabolomic changes but might be still reversible^31^, suggesting high phenotypic plasticity rather than local adaptation. This transition is also dependent on the host interactions with its complex bacterial communities^29^. Although *E. subulatus* is a different species, such host-bacterial interactions may also play a role in the adaptation of *Ectocarpus* sp. to its environment (although this aspect has not been investigated in the study presented here).

Analysis of the *Ectocarpus* sp. genome suggested that this species has evolved effective mechanisms to survive its harsh intertidal environment such as a complex photosynthetic system and a large number of ion channels^1^. These characteristics may have contributed to its ability to colonize freshwater environments. However, even with the current improved assembly and annotation of the *Ectocarpus* genome, about 39% of the predicted genes lack functional annotations based on available databases^32^. Hence, an important proportion of the genes in the genome do not have a predicted function and, therefore, approaches such as detection of quantitative trait loci (QTLs) and analyses of polymorphism of candidate genes^33^ are important to explore the genetic basis of adaptation in this species.

QTL analysis provides insights into the genetic determinism of traits and is an important aid for marker assisted selection^34^,^35^. QTL mapping has been used successfully to improve our understanding of the genetic basis of traits in land plants^36^ and in animals, including marine animals^37^, but remains rarely investigated in algae^38^. With recent advances in sequencing technology, molecular markers such as single nucleotide polymorphisms (SNPs) can now be identified in abundance at continually decreasing cost, even in complex genomes. Methods such as Restriction site Associated DNA (RAD) sequencing allow the complexity of the genome to be reduced in order to produce high confidence SNPs cost effectively for various applications^39^,^40^.

In our study, we generated 3,588 RAD-seq-based SNP markers to construct a dense genetic map for *Ectocarpus* sp. and used it to identify QTLs for growth-related traits and their plasticity under different temperature and salinity conditions. Several candidate QTLs for high temperature and low salinity tolerance were detected and GO (Gene Ontology) enrichment tests within these QTLs contributed to our understanding of the ability of *Ectocarpus* sp. to adapt to high temperature or low salinity.

## Results

### Identification of phenotypic traits under temperature and salinity stress

To study the genetic basis of growth under temperature and salinity stresses, we grew a family of 89 *Ectocarpus* sp. haploid progeny, together with their two parental strains (six clones for each strain) for 12 days in growth cabinets under three temperature (13 °C as control, 26 °C and 28 °C; hereafter T13, T26 and T28) and three salinity conditions (34‰ as control, 20‰ and 15‰; hereafter S34, S20 and S15). Temperature and salinity stresses were applied under normal, control salinity (34‰) and temperature (13 °C) conditions, respectively. An initial series of test experiments aimed at selecting optimal temperature and salinity conditions for QTL detection had shown that 30 °C and 10‰ salinity were extreme conditions for the mapping family, leading to high mortality. We therefore selected our stress conditions so that they had significant impacts on the growth phenotype of the offspring without killing a high proportion of individuals (we considered up to 30% of mortality as acceptable, i.e. none of the conditions should induce a complete loss of more than 26 progeny).

Growth, expressed as a ratio of the thallus area on day 12 to the thallus area on day 0, varied significantly across the studied progeny under all three salinity conditions (p-value between 4 × 10^−4^ and 7 × 10^−16^, ANOVA) but only under two of the three temperature conditions (p-value = 0.005 for 26 °C, p–value < 1 × 10^−10^ for 28 °C and p = 0.2 for 13 °C, ANOVA). A significant sex-dependent difference was observed under 15‰ salinity (adjusted p-value Tukey comparison test: 3 × 10^−7^) with males showing more growth than females (growth ratio of 0.47 vs 0.30 respectively).

**Figure 1:**
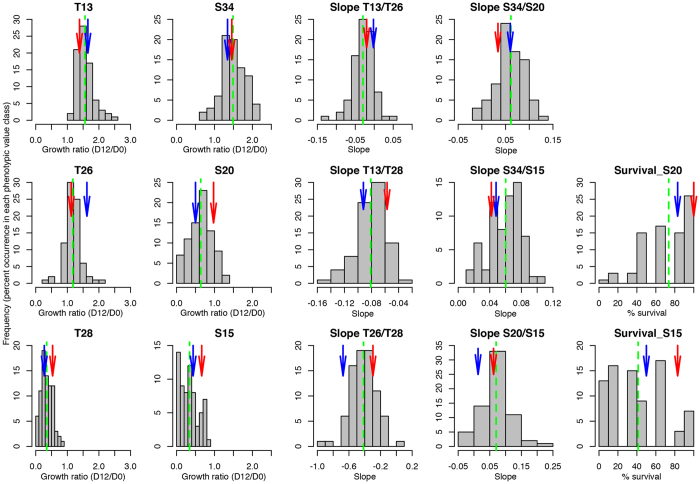
Distributions of the progeny BLUPs of the studied traits. T13, T26 and T28 correspond to growth rate phenotypes for temperatures 13 °C, 26 °C and 28 °C respectively. S34, S20 and S15 correspond to growth rate phenotypes for 34‰ salinity, 20‰ and 15‰ respectively. Plasticity traits are represented by a letter followed by a number and a slash, then another letter and number. For example, S34/S20 corresponds to plasticity represented by slope of the reaction norm between salinity 34‰ condition and salinity 20‰ condition. Surv_S15 and Surv_S20 correspond to survival trait for conditions salinity 15‰ and salinity 20‰. Frequency distributions of the progeny BLUPs of the analyzed traits; blue arrows indicate mean values for the male parent and red arrows indicate mean values for the female parent; the green dashed line represents the mean value for the progeny population; “Slope” represents the slope values of the reaction norm between two conditions indicated at the top of each graph; “% survival” represents the proportion of survival among replicates of each progeny for salinity conditions 15‰ and 20‰.

The impact of the applied stresses was clearly visible through their effects on the distribution of the traits, with a reduced growth rate for the stress conditions and a much lower population mean for the growth ratio (Fig. 1). We observed substantial variance in all traits across the progeny, with the fastest growers having growth ratios 2 to 5-fold larger than the slowest growers. Differences between the mean values for each parent were more limited.

Transgressive individuals (i.e. displaying more extreme phenotypes than the parental strains) were observed in the mapping family under almost all conditions (Fig. 1).

By treating different experimental conditions as different environments, we observed significant genotype by environment (G × E) interactions (genotype × salinity p-value = 2 × 10^−6^ and genotype × temperature p-value = 4 × 10^−4^), which is potentially indicative of a genetic basis of variation of phenotypic plasticity^41^,^42^. Reaction norms of the progeny for temperature and salinity variations were also analyzed. The results showed that several reaction norms had different slopes and therefore crossed, suggesting significant G × E (Supplementary Fig. S1). We therefore used the slopes of these reaction norms as plasticity traits. There were three temperature plasticity traits (T13/T26, T13/T28 and T26/T28) and three salinity plasticity traits (S34/S20, S34/S15 and S20/S15, Fig. 1).

In total, 15 traits were tested: 3 for temperature, 3 for salinity, 3 for temperature plasticity, 3 for salinity plasticity, 2 for survival, plus sex (Fig. 1).

Broad-sense heritability estimates for growth related-traits and survival varied between 0.20 and 0.78 for individual traits and between 0.10 and 0.72 when combining all conditions of temperature and salinity (Table 1).

### Construction of a ddRAD-seq-based genetic map

Barcoded ddRAD libraries were constructed for the two parents and their 89 progeny using the enzymes *Pst*I and *Hha*I and were pooled for sequencing. Of the initial total of 470 million reads, the Process_radtags module of the Stacks pipeline together with additional cleaning steps retained 339 million reads. The Ref_map process in Stacks generated 183,336 consensus sequences from this sequence data, of which 181,655 were unique, and the Genotypes program identified 11,740 putative markers within these regions. After application of a series of filters (see materials and methods), 6,275 of the 11,740 markers identified by Stacks were retained. During the filtering, nine individuals (out of the 89 progeny) with excessive missing genotypes were removed.

For the construction of the genetic map, distorted markers that did not show the expected 1:1 segregation pattern (at a threshold of 0.05%) were also filtered out. When two or more markers exhibited similar segregation patterns (based on a similarity of ≥0.95), only one of the markers was retained. Markers with suspect linkages were also filtered out. Finally, a total of 3,588 markers and 80 individuals were used for the construction of the genetic map. Maps obtained with R/qtl and JoinMap using this data were compared and found to be very similar.

The 3,588 SNPs were distributed over 28 linkage groups (LGs), with a minimum logarithm of the odd (LOD) score of 4.0. The total length of the map was 2,585.7 centimorgans (cM), which accounted for 98.35% of the estimated genome (Ge) length (Ge = 2629 cM). The average spacing between two adjacent markers was 0.7 cM and the largest gap was 20.3 cM (on LG28). The lengths of the 28 LGs ranged from 41.8 cM with 54 markers to 152.3 with 217 markers (Table 2, Supplementary Fig. S2 and Supplementary Dataset).

Considering the 214 Mbp genome size of *Ectocarpus* sp.^1^, the estimated global recombination rate was 12.28 cM/Mb. The average recombination rate per LG varied between 11.11 cM/Mb for LG28 and 17.93 cM/Mb for LG4. At a 1 Mbp scale along the different LGs, the recombination rate varied considerably with broad patterns of decreases and increases (Supplementary Fig. S3). However, there were no substantial differences between the LGs.

### Identification of QTLs for responses to temperature and salinity stresses

**Figure 2:**
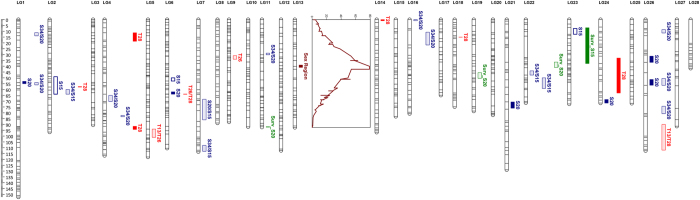
Genetic map showing the localization of detected QTLs for temperature, salinity and their associated plasticity and survival traits. Blue color indicates salinity and its plasticity traits, red indicates temperature and associate plasticity and green indicates survival traits. The brown color on LG13 represents the sex-determining region and the QTL LOD score curve from a Kruskal-Wallis non-parametric test in MapQTL. T13, T26 and T28 correspond to growth rate phenotypes for temperatures 13 °C, 26 °C and 28 °C respectively. S34, S20 and S15 correspond to growth rate phenotypes for 34‰, 20‰ and 15‰ salinity, respectively. Plasticity traits are represented by a letter followed by a number and a slash, then another letter and number. For example, S34/S20 corresponds to plasticity represented by the slope of the reaction norm between 34‰ and 20‰ salinity. Surv_S15 and Surv_S20 correspond to survival traits for 15‰ and 20‰ salinity, respectively.

Combination of genotyping and phenotyping data allowed the identification of a total of 39 QTLs, with between 0 and 10 QTLs detected per trait across a total of 15 traits tested. The highest number of QTLs (10) was observed for the plasticity trait S34/S20. No QTLs were detected for the traits T13 and S34. Each QTL is associated among others with the observed phenotypic variance (*R*^2^) and an estimated additive effect (*a*) that it accounts for. The detected QTLs explained 6% to 34% of the observed phenotypic variance, depending on the trait (Table 3). In some cases, QTLs for different traits mapped approximately to the same genomic region (Fig. 2). For all the temperature and salinity traits (i.e. growth ratios), and for the plasticity traits, there were both cases where the allele transmitted by the male parent was favorable (increasing the growth ratio) and cases where it was unfavorable (decreasing the growth ratio), indicating antagonistic QTLs (see values of additive effect *a* in Table 3). In contrast, for the two survival traits, the male allele was always unfavorable (lowering the survival value).

The sex of each individual was determined using PCR primers based on the coding sequence of the sex-linked gene Esi0068_0003^21^,^43^. Using this characteristic as a binary trait, a major sex-associated locus was mapped to LG13. This result confirmed the position of the previously identified sex-determining region^21^.

Twenty-one significant epistatic interactions were detected, involving 38 unique linkage map positions. Among these interactions, only six involved previously detected QTL positions (Supplementary Table S1). For example, in the case of trait T28, the result of epistasis between LG1 and LG14 (Supplementary Fig. S4) showed that the locus on LG1 (at 6 cM) had an effect only in the presence of the B genotype (A and B genotypes derived from the male and female parents, respectively) at the locus on LG14 (at 64 cM). In the presence of the A genotype at the locus on LG14, no significant difference was observed between the growth ratio of individuals carrying the A genotype and those carrying the B genotype at the locus on LG1.

We also detected five significant QTL × sex interactions, where the effect of the locus was different for the two sexes, representing potential sexually antagonistic alleles (Supplementary Fig. S5).

### Gene ontology enrichment test

A genome-wide list of candidate genes was constructed by retaining all loci in the 1.5-LOD support confidence intervals of the complete set of QTLs. This list of 562 genes included 263 genes that had associated GO terms. In the re-annotated genome of *Ectocarpus*^32^, 17,418 genes were identified among which 10,688 had predicted functions and 7,383 had associated GO terms. We built a reference list, consisting of genes located in 10 kb windows around the SNPs localized on the genetic map. This gave a list of 2,710 genes among which, 1,156 had associated GO terms (Supplementary Dataset). A GO enrichment test was carried out by comparing the candidate list of 263 genes which had associated GO terms with the reference list of 1,156 genes also having associated GO terms. Six terms were significantly enriched in the candidate gene list compared with the reference at *p* = 0.05, with one term having a p-value < 0.01. Four of those 6 terms were linked to transport processes, the most significant being anion transport (Table 4, Supplementary Fig. S6 and Supplementary Dataset).

## Discussion

Growth under different temperature and salinity conditions showed a distribution with the presence of transgressive segregants among the offspring in almost all cases, even when there was very little difference between the parents. Transgressive individuals may have characteristics allowing them to occupy new ecological habitats or to perform more effectively in already occupied environments^44^. These transgressive segregants are likely to be the result of complementary action of different alleles of genes from the male and female parent^45^,^46^ and their presence provides the potential for improvement of the traits using selective breeding or as a result of natural selection.

**Figure 3:**
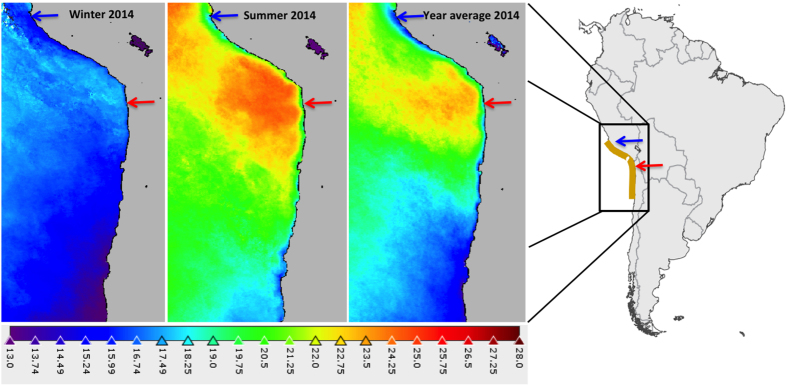
Sea surface temperature (sst) in the distribution range of *Ectocarpus* sp. for year 2014. The blue arrow represents the original location of the male parent (San Juan de Marcona, Peru) and the red arrow represents the original location of the female parent (Arica, Chile). Created with the SeaWiFS Data Analysis System (SeaDAS) visualization and data processing software version 7.2, using seasonal (winter and summer) and annual MODIS sst data from 2014: http://seadas.gsfc.nasa.gov/installers/; http://oceandata.sci.gsfc.nasa.gov/MODIS-Aqua/Mapped. NASA Goddard Space Flight Center, Ocean Biology Processing Group; (2014): Sea-viewing Wide Field-of-view Sensor (SeaWiFS) Ocean Color Data, NASA OB.DAAC, Greenbelt, MD, USA. http://doi.org/10.5067/ORBVIEW-2/SEAWIFS_OC.2014.0. Accessed 2015/05/11. Maintained by NASA Ocean Biology Distibuted Active Archive Center (OB.DAAC), Goddard Space Flight Center, Greenbelt, MD, USA.

The two parents of the mapping family originated from the west coast of South America, between the south of Peru and the north of Chile, representing about 2,000 km of coastal ecosystem where seasonal variation in seawater temperature is high (between 13 °C in winter and 25 °C in summer on average, Fig. 3). The parental strains are therefore presumably adapted to living in a changing environment. The annual average temperature of the original location of the male parent is lower than that of the female parent (Fig. 3). And it is noteworthy that it was only at the highest temperature that the female parent performed better than the male one. This may be explained by the mean temperatures at the parental locations, but since 28 °C is not a common mean temperature encountered there, this is rather speculative. Nevertheless, their occurrence in rockpools or low tides during summer months can expose these *Ectocarpus* strains to very high temperatures. Furthermore, the average seasonal temperature of seawater in the distribution zone of the species can increase by 4 °C to 10 °C during *El Niño*-Southern Oscillation events^47^.

The distributions of growth ratios varied little at 13 °C and 26 °C but significantly decreased at 28 °C. Even at 28 °C, the majority of the offspring could maintain non-null growth. A temperature of 30 °C may exceed the tolerance of this family as observed during preliminary tests, at least under our laboratory conditions, but it is possible that the response of the population to extreme temperatures would show some differences under field conditions compared to the observations in laboratory conditions, particularly because several stressors act in synergy in the wild.

For salinity stress, the female parent always performed better than the male parent. Typical salinity of seawater in the distribution range of the species is 34–36‰. However, our data showed that several strains were able to maintain active growth even at 15‰. Although most of the species of the *Ectocarpus* genera are predominantly marine, some strains (e.g. *E. subulatus* isolates) are tolerant to low salinity (i.e. occur in estuaries) or are even adapted to a freshwater environment^31^,^48^. The maintenance of a capacity to respond to large variations in water salinity in the extremely arid region of the Atacama Desert, where there are no estuaries, is likely a result of an adaptive evolution in other regions of the coast. Interestingly, this low salinity tolerance in *Ectocarpus* species may explain why there are cases of colonization of freshwater environments, albeit rare. Dittami *et al*.^31^ suggested that, while high stress tolerance and plasticity may be prerequisites for the colonization of freshwater, genomic alterations are likely to have occurred and these alterations would have produced permanent changes in metabolite profiles to stabilize the transition.

Broad-sense heritability estimates were low to moderate but increased with the degree of stress for the environmental conditions we tested (from 0.20 to 0.78). The heritability estimates were lower when temperature and salinity stress were combined (0.10 and 0.31). It is well known that estimated heritability of a trait can vary either with environmental conditions or between populations. Hoffmann and Merilä^49^ summarized hypotheses to explain the relationship between environmental effects and heritability. It is possible that phenotypic variations between genotypes would be difficult to detect unless resources become limiting, regardless of the history of selection^50^. In contrast, unfavorable conditions could also decrease the importance of observed genetic variation if increased environmental variance reduces the adaptive value of individual genotypic combinations. This might apply to our study, when we combined stress conditions for temperature and for salinity, hence increasing the overall environmental heterogeneity. Furthermore, although we were able to replicate genotypes in this studied species, traits are nonetheless measured with some error and this might lead to an underestimate of heritability if phenotypic variance is higher under unfavorable conditions^51^.

With 3,588 SNP markers, the genetic map described here is considerably denser than the previously published map for *Ectocarpus* sp., which was based on 406 microsatellite markers^20^. Overall, the distribution of the markers on the 28 LG was homogenous. The 28 LGs detected here are coherent with cytogenetic studies carried out using European strains of *E. siliculosus* that indicated an approximate number of 25 chromosomes^52^.

Assignation of the SNP markers to sequence scaffolds allowed 500 of the 1,561 scaffolds of the genome sequence to be anchored onto linkage groups, significantly improving the large-scale assembly of the genome^32^. Integration of this new information along with the data from the microsatellite-based linkage map^20^ has allowed a total of 531 scaffolds to be anchored onto linkage groups, corresponding to 177 Mbp of sequence data and 90.5% of the total genome. The improved genetic map will represent an important resource for future genetic and comparative genomic studies using *Ectocarpus* sp. as a model species.

The quality of a genetic map is influenced by the density of the markers used and the size of the mapping family. As far as marker density is concerned, next generation sequencing technologies represent a means to tremendously improve the generation of genetic markers and the ddRAD sequencing approach used in this study produced enough markers to cover the major part of the genome. However, the mapping family is a “pseudo F2” haploid population where each individual is derived from a separate but unique meiosis. This, plus the relatively small size of the family, constitute factors that may limit the power of QTL detection^53^.

We estimated a global recombination rate of 12.28 cM/Mb for *Ectocarpus* sp., an estimate that is higher than values obtained for most terrestrial plant species^54^. If we examine data available for *Saccharina japonica*^55^,^56^, the only other brown algal species for which both a genetic map and a complete genome sequence currently exist, the global recombination rate is 4.93 cM/Mb. This value is more similar to those observed for angiosperms. The genome size of *Saccharina japonica* is 537 cM^56^, which is 2.5 times larger than that of *Ectocarpus* sp. These estimates are in accordance with the observation of negative correlation between global recombination rate and genome size, pointing to a putative role of LTR retrotransposons^54^. More estimates are needed for additional brown algae to provide improved insights into global recombination rates in this group. In any case, recombination is a very complex feature because it is known to vary substantially across eukaryote genomes and even between individuals within populations, for reasons that are still to be fully understood^57^. In our study, recombination rates varied along and between the different *Ectocarpus* sp. LGs (average rate per LG between 11.11 cM/Mb for LG28 and 17.93 cM/Mb for LG4), at a 1 Mb scale. Although no clear differential patterns between the LGs were observed, this result should be considered with caution for several reasons. First, resolution of cross-based estimates of recombination rates like those we provide here depends on the number of recombination events occurring in a generation, and the generation of the family analyzed here involved only one meiosis per individual. Second, because the scale at which the recombination rates are examined may influence the detected relationships between recombination and genomic features and, consequently, patterns at coarse scales may not accurately reflect relationships at finer scales^58^.

Our experimental setup allowed us to detect 38 QTLs for growth-related traits and their plasticity under different temperature and salinity conditions and 1 QTL for sex. Eighteen of the 28 LGs contained QTLs for these traits. Although several of the QTLs had large confidence intervals, some showed relatively small ones. For example, the QTLs for T28 on LG2 and LG18 had small confidence intervals, which contained respectively 41 and 6 annotated or predicted genes. There was only one case of co-localization of QTLs (for temperature and salinity on LG2 for traits T28 and S15). The rarity of co-localization of QTLs was in agreement with the low to moderate correlation between traits, with most correlations being statistically non-significant (Table 5). More co-localizations were observed when survival and plasticity traits were considered. In three cases (LG1, LG2 and LG26), plasticity QTLs co-localized with main traits. These co-localizations could be due to the presence of genes that control metabolic pathways that influence multiple traits or to the presence of pleiotropic genes.

Phenotypic plasticity is one of the strategies used by organisms to cope with variability in their environment but its genetic basis is often poorly documented^59^. Plasticity often involves altering gene expression and physiology in response to environmental cues^60^. The QTL approach developed here could allow evolutionary questions such as the evolution of the reaction norm as a response to the changing environment to be addressed. The detection of plasticity QTLs in *Ectocarpus* sp. indicates that factors controlling plasticity in this species are likely to be genetically-based^41^. We also identified several antagonistic QTLs (i.e. QTLs with alleles that have opposing effects). It is thought that, in such cases, trait differences may have arisen through drift and/or through selection that fluctuates in direction^46^,^61^. Antagonistic QTLs are also considered to be able to generate transgressive segregants^46^, which is in accordance with our results.

Epistasis is considered to be an important genetic basis of complex phenotypes^62^,^63^. More than one trait was involved in all the 21 cases of epistatic interactions detected in this study, suggesting pleiotropy not only for single loci but also for epistatic effects. Among those 21 epistatic interactions, we observed only six cases where one of the markers had a main QTL effect on the trait. This suggests that epistasis is an important mechanism for the control of the response to stress in *Ectocarpus* sp., at least when growth ratio is used as a proxy for fitness.

Few QTL × sex interactions were identified in our study. These interactions were sex-antagonistic since the effects of the QTL alleles were opposite for the two sexes. Such interactions indicate that the effect of a QTL is different between the two sexes and this can be important in evolutionary as well as in breeding contexts. Evolutionary arguments implicate QTL × sex interactions in the maintenance of variation for complex traits^64^. From a biological point of view, QTL × sex interactions can be explained by invoking interactions between genes with different effects in different sexual environments (considering sexes as different environments and hence QTL × sex interactions to be QTL × environment interactions)^65^. However, we cannot exclude the possibility that the observed QTL × sex interactions observed in our study may just reflect the different origins of the parental strains and their specific trait inheritance, since we cannot separate the effects of their sex and their origin. The detection of main effects, epistasis and sexually-dimorphic QTLs suggest complex genetic regulation of growth under stress conditions.

A GO enrichment test identified six terms significantly enriched in putative genes lying in the QTL intervals, among which, four were linked to ion and protein transport processes. Of the 562 putative genes in the QTL intervals, 102 were also identified as being significantly differentially expressed between low and high salinity by Dittami *et al*.^31^ (Supplementary Dataset), but more interestingly, the authors also observed an accumulation of intracellular ions at different levels between freshwater and marine strains, suggesting an important role for ion transport in the adaptation to low salinities. The list of putative stress-related genes corresponding to the QTL intervals identified in the current study should be treated with caution because there are uncertainties linked to positions of QTLs and because growth is a complex phenotype integrating numerous metabolic pathways. Taking into account these limitations, the data presented here is however in accordance both with the suggested importance of intracellular ion composition and osmotic balance in adaptation to stresses such as low salinity and with the large number of ion channels observed in the genome of the species^1^,^31^. Furthermore, transport processes such as ion transport are well studied in land plants and have been shown to be involved in the response to osmotic stresses^16^,^66^. In seagrasses, several genes have been also shown to respond to elevated sea surface temperatures^67^ or to be under selection along the salinity gradient of the intertidal shore^68^.

In our study, we built a high-density genetic map using ddRAD-seq approaches and detected several QTLs for growth rate under high temperature and low salinity stresses as well as related plasticity and survival traits. GO enrichment tests suggested a role for ion transport genes among the main loci controlling the QTLs. This study represents a significant step towards deciphering the genetic architecture of adaptation of *Ectocarpus* sp. to stress conditions and, furthermore, adds a substantial resource to the increasing list of resources generated for the species. It also opens new perspectives in population genomics of adaptation in brown algal species. As mentioned in the introduction, brown algae have also been shown to display important interactions with complex bacterial communities, which influence their ability to respond to environmental stressors and this aspect makes the picture more complex. Several studies have shown that algal-bacterial associations are beneficial for the algal hosts, increasing their fitness^69^,^70^ and some brown algal species such as *Ectocarpus fasciculatus* and *Pylaiella littoralis* are absolutely dependent on their associated bacteria^71^,^72^. These complex interactions were not included in this study but should be examined in future studies in order to have a more complete understanding of the adaptation of *Ectocarpus* sp. to its stressful environments.

## Materials and Methods

### Mapping family

The mapping family was generated by crossing the male *Ectocarpus* sp. strain Ec 32 (whose genome has been fully sequenced) with a compatible female strain, Ec 568. See Heesch *et al*.^20^ for a detailed description of the production of the family. Briefly, a single F1 hybrid sporophyte isolated from the cross (Ec 569) was used to produce a family of sibling haploid gametophytes, each individual being derived from a separate meiotic event. Gametes from each individual were then allowed to germinate parthenogenetically to produce haploid partheno-sporophytes. Eighty-nine progeny of this haploid family, plus haploid partheno-sporophytes derived from the parental gametophytes, were cultivated for the experiments.

### Culture conditions and phenotyping

For both the temperature and salinity stress experiments, preliminary tests were carried out to determine conditions that resulted in phenotypic differences without causing extreme mortality. Three temperature conditions were selected: 13 °C (control condition) and two higher stress temperatures, 26 °C and 28 °C. Similarly, three salinity conditions were also selected: 34‰ (normal sea salinity) as the control and two low salinity conditions, 20‰ and 15‰. For each strain, 6 replicates were used and individuals were randomly distributed in 6-well plates containing 6 ml of autoclaved, Provasoli-enriched seawater. Natural seawater was used for temperature experiments (salinity is stable at around 33.5‰ to 34.5‰) while artificial seawater was used for salinity experiments. Note that differences might exist between natural and artificial seawater regarding nutrients. Experiments were carried out in growth chambers and the positions of the plates were permutated daily to reduce microenvironment effects. Light conditions were 12 h/12 h day/night (~2000 lux light intensity) and the temperature in the growth chamber was set to 13 °C for the salinity experiment. The plates were maintained in the growth chambers for 12 days. Each individual was photographed under a binocular microscope before and after the experiment and thallus area was measured using the program ImageJ. Growth was estimated as the ratio of the thallus area on day 12 to the thallus area on day 0. By treating different experimental conditions as different environments, we determined reaction norms of the mapping progeny and used the slopes of these reaction norms as plasticity traits. The relation of these plasticity traits to specific environmental variations was indicated by giving the two environmental conditions separated by a slash mark (e.g.: S34/S20 is the plasticity trait between salinity conditions 34‰ and 20‰).

### DNA extraction and generation of RAD-seq data

For DNA extraction, the haploid family together with the parental lines were grown in a growth chamber in sterile seawater medium for 6 weeks under a 12-hour light photoperiod and 13 °C. As the cultures were not fully axenic, 5 ml of a mix of antibiotics (9 mg/ml of Penicillin G, 4.5 mg/ml of Streptomycin and 0.9 mg/ml of chloramphenicol) were added to each liter of seawater used for the culture to reduce the bacterial load. Harvested individuals were frozen and lyophilized, and DNA was extracted using the NucleoSpin^®^ 96 Plant II kit (Macherey-Nagel GmbH & Co. KG, Germany), according to the manufacturer’s instructions. DNA quality was checked on agarose gels and the quantity measured by PicoGreen^®^ (Fisher Scientific).

Preparation of the double digest RAD library (ddRAD-seq) was carried out following Peterson *et al*.^40^. The genomic DNA was digested with the restriction enzymes *Pst*I and *Hha*I (New England Biolabs, https://www.neb.com/), which had been selected based on *in silico* digestion simulations, and adapters were ligated using the T4 DNA ligase (New England Biolabs, https://www.neb.com/). After pooling the samples which had been individually barcoded with a unique adapter and cleaning with AMPure XP beads (Beckman Coulter Genomics), size selection (between 150 and 600 bp) was carried out with a Pippin-Prep kit (Sage Science, Beverly, MA, USA). A PCR amplification was then carried out using the Q5^®^ hot Start High-Fidelity DNA polymerase kit (New England Biolabs) to increase the concentration of the libraries and to add Illumina flowcell annealing sequences, multiplexing indices and sequencing primer annealing regions, to all fragments. Compared with the original protocol, the barcodes were modified by adding an extra nucleotide, extending to six nucleotides (see Supplementary Dataset). Both an Agilent^*®*^ 2100 Bioanalyzer^®^ (Agilent Technologies) and qPCR were used to quantify the libraries generated from each individual. Libraries with distinct multiplexing indices were then combined in equimolar ratios to compose a final pool of libraries for sequencing. An Illumina Hiseq 2500 platform (Rapid Run Mode) was used to generate a total of 470 million (48.5 Go) high quality (Q30 = 82%) 100 bp paired-end sequence reads at a cluster density of 850 K/mm^2^ (HiSeq Rapid SBS Kit v2, 200 cycles). As a test, a subset of the library was also sequenced with Illumina MiSeq, providing 93 million additional reads (MiSeq Reagent Kit v3, 150 cycles) The raw sequence data in fastq format are stored in the Sequence Read Archive (SRA) at National Center for Biotechnology Information (NCBI) and can be accessed at NCBI homepage (https://www.ncbi.nlm.nih.gov/; bioproject accession PRJNA371840 or study accession SRP099384).

### Analysis of ddRAD-seq data

The ddRAD-seq sequencing data was analyzed using the Stacks pipeline (version 1.40)^73^. Raw sequence reads were checked for intact barcodes and the restriction enzyme sites. A sliding window of 25% of the length of a read was set to check sequence quality and reads with <90% base call accuracy were discarded. Because Stacks removes whole reads when adapter contamination is detected, we first used the program Cutadapt (version 1.8.3) to check for the presence of adapters and cut only adapter sequences. Paired-end sequencing of shorter fragments generated overlapping reads that were treated with the program PEAR (version 0.9.10)^74^. PEAR identifies paired-end non-overlapping reads and generates a single consensus sequence from overlapping read pairs. These single consensus sequences were added to the singleton rem1 and rem2 sequences produced by Stacks for a unique group of singleton sequences. Paired-end reads as well as singletons were then trimmed to 95 bp with the program TRIMMOMATIC (version 0.32)^75^. The paired-end sequences and the singletons were then aligned to the *Ectocarpus* reference genome^1^ using Bowtie 2 (version 0.12.9)^76^. Output aligned. sam files were subsequently imported in to the Stacks pipeline and the final Genotypes program exported haplotypes encoded as genotypes at the different loci.

### Construction of the genetic map

After Stacks analysis, genotypes were exported in a generic format that was imported into Excel for filtering. As we used a haploid population, only aa/bb segregation was expected. All non-expected segregation patterns, as well as loci with log likelihood values below −30, were removed. Loci with over 25% of missing data were also removed. In the finale matrix (see Supplementary Dataset), A genotypes derived from the male parent and B genotypes derived from the female parent. The R package R/qtl (version 1.39–5)^77^ was used for the construction of the genetic map, in conjunction with either R/ASMap (version 0.4–7) or JoinMap^®^ version 4.1. At this stage, individuals with more than 40% of missing genotypes were removed. Further filtering was done within the mapping programs. Markers that showed significant segregation distortion (≤5%) were removed. Recombination fraction estimates were used to correct erroneous genotypes or switched alleles. Markers were also filtered based on their similarity, on whether they were located on the same contig and based on suspect linkage patterns. In JoinMap, the maximum likelihood mapping algorithm was applied.

Estimated genome (Ge) length was obtained by adding 2 s (s being the average spacing between markers along the LGs) to the length of each LG to account for chromosome ends beyond the terminal markers of each LG^78^. The map coverage was then estimated as the ratio between the observed map length and Ge.

The R/xoi package (version 0.66–9) was used to obtain a smoothed estimate of the recombination rate along the LGs, in 1 Mbp sliding windows.

### Statistical analyses of phenotypic data

Statistical treatments were done in R. BLUPs were obtained by fitting several linear mixed models (LMM) with the R package lme4 (version 1.1–12) and selecting the optimal model with AIC:


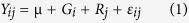


where *Y*_*ij*_ represents the value of the trait under investigation for the genotype *i* at replicate *j, μ* the general mean of the trait, *G*_*i*_ the random effect of the genotype *i, R*_*j*_ the random effect of the replicate *j* and *ε*_*ij*_ the random residual effect.

Broad-sense heritability of individual traits was calculated as:


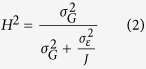


where 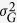 is the genetic variance, 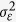 the environmental variance and *J* the number of replicates.

Some strains did not survive at salinities of 15‰ and 20‰ and hence we introduced a binomial survival trait. The survival traits were analyzed by fitting generalized linear mixed-effects models (GLMM) and using 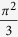 as an approximate to the environmental variance as suggested by Nakagawa and Schielzeth^79^.

The different temperature, salinity or survival conditions were also combined as a single phenotype (temperature, salinity or survival). Variances and broad-sense heritabilities were obtained by fitting LMMs or GLMMs and selecting the optimal model:


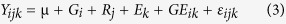


where *E*_*k*_ is the fixed effect of environment (different conditions of temperature, salinity or survival) and *G*_*ik*_ the random interaction effect between genotype *i* and environment *k*. The sex of each strain was included in the data. Broad-sense heritability of individual traits was calculated as:


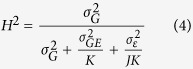


where 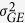 is the G × E interaction variance, *K* the number of environments and *J* the number of replicates.

### QTL mapping

QTL mapping was performed using the R package R/qtl^77^ as well as MapQTL^®^ version 5. In R/qtl, the scanone function was used to perform interval mapping with the Haley-Knott regression method, including sex as a cofactor. 5% significance thresholds of the LOD scores were obtained by 5,000 permutations. In MapQTL, composite interval mapping was performed with the MQM method. The method uses markers as cofactors in an approximate multiple-QTL model with additive and dominant gene actions. Cofactors were selected in a first round as loci with LOD ≥2.5 after a simple interval mapping. Then this list was extended with markers on a given linkage group selected every 10 or 20 cM and optimized via the automatic cofactor selection method. The method uses a backward elimination procedure to determine which markers show a significant association and which do not, and ultimately produces a list of significant cofactor markers. For survival traits and sex, the “binary” or the “nonparametric” models were applied. BLUP values were used for QTL mapping. For QTL confidence intervals, 1.5-LOD supports were used. Mapchart 2.3^80^ was used to draw the QTL on the linkage map.

QTL × sex interactions were detected by using “sex” as a covariate. QTL × QTL additive epistatic interactions were detected by the scantwo function of R/qtl, performed by the Haley-Knott regression. Four models were used for this: a full model where the two QTLs are allowed to interact, an additive model where the QTLs are assumed to act additively, a single-QTL model, which corresponds to the result of the single-QTL genome scan and a null model assuming no QTL^81^.

### Gene ontology enrichment test

Taking advantage of the fact that the genome of the species has been sequenced and carefully annotated, we tested GO enrichment among the putative genes within the QTL intervals. For that, we made two lists: a candidate list consisting of all loci in the 1.5-LOD support confidence intervals of the QTLs, based on the sequenced reference genome and a reference list including all the putative genes in the mapped scaffolds, refining the search only on a 10 Kb window around each SNP on the genetic map. Only loci with clear GO identifications were retained from the two lists. The candidate list was then compared to the reference list using the R/topGO package (version 2.26.0), which is designed to facilitate semi-automated enrichment analysis for GO terms. The test was based on gene count and used the “classic” algorithm with the “Fisher” statistic.

## Additional Information

**How to cite this article**: Avia, K. *et al*. High-density genetic map and identification of QTLs for responses to temperature and salinity stresses in the model brown alga *Ectocarpus. Sci. Rep.* **7**, 43241; doi: 10.1038/srep43241 (2017).

**Publisher's note:** Springer Nature remains neutral with regard to jurisdictional claims in published maps and institutional affiliations.

## Supplementary Material

Supplementary Information

Supplementary Dataset

## Figures and Tables

**Table 1:** Trait statistics.

Trait	Mean	Min	Max	*V*_*g*_	*CV*_*g*_	*H*^*2*^ (*s*.*e*.)
S34	1.49	0.67	2.16	0.039	0.13	0.42 (0.11)
S20	0.64	0.024	1.3	0.054	0.36	0.78 (0.04)
S15	0.37	0.008	0.9	0.033	0.49	0.78 (0.05)
T13	1.56	1.17	2.42	0.014	0.08	0.20 (0.14)
T26	1.18	0.4	2.07	0.024	0.13	0.36 (0.14)
T28	0.34	0.03	0.86	0.019	0.41	0.62 (0.08)
Surv_S20	—	—	—	1.02	—	0.65 (0.09)
Surv_S15	—	—	—	1.73	—	0.76 (0.06)
Salinity	1	0.008	2.16	0.009	0.09	0.31 (0.19)
Temperature	1.03	0.03	2.42	0.002	0.04	0.1 (0.18)
Survival	—	—	—	1.42	—	0.72 (0.05)

T13, T26 and T28 correspond to growth rate phenotypes for temperatures 13 °C, 26 °C and 28 °C respectively. S34, S20 and S15 correspond to growth rate phenotypes for 34‰ salinity, 20‰ and 15‰ respectively. Surv_S15 and Surv_S20 correspond to survival trait for conditions salinity 15‰ and salinity 20‰. Salinity, Temperature and Survival correspond to the joint analysis of the three salinity conditions, the three temperature conditions and the two survival conditions respectively. *V*_*g*_ and *CV*_*g*_ corresponds to genetic variances and their coefficients of variation (*CV*_*g*_ expressed as 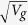/trait mean), *H*^*2*^ (*s*.*e*.) corresponds to broad-sense heritabilities and their standard errors estimated via jackknife resampling. Min and Max are based on average values for individuals and not the raw replicate values.

**Table 2:** Linkage group statistics for the genetic map.

LG	Number of markers	LG length (cM)	Average spacing between markers (cM)	Maximum spacing between markers (cM)
1	217	152.3	0.7	11.2
2	194	96.5	0.5	5.3
3	202	90.4	0.4	8.5
4	180	116.7	0.7	7.6
5	168	117.9	0.7	6.7
6	171	110.4	0.6	8.8
7	170	113.4	0.7	9.3
8	152	88.7	0.6	6.3
9	155	87.8	0.6	7.6
10	138	92	0.7	8
11	120	92	0.8	10
12	127	112.4	0.9	8.9
13	124	92.6	0.8	7.6
14	124	96.7	0.8	6.3
15	119	83.1	0.7	7.8
16	106	80.7	0.8	12.8
17	111	64.9	0.6	7.6
18	116	74.8	0.7	11.5
19	107	78.5	0.7	8.7
20	112	81.8	0.7	10.1
21	87	128.9	1.5	12.8
22	103	71.4	0.7	18.3
23	91	72.4	0.8	8.8
24	90	71.6	0.8	8.8
25	96	71.9	0.8	7
26	79	112.5	1.4	16.9
27	75	91.5	1.2	19.4
28	54	41.8	0.8	20.3
**overall**	**3588**	**2585**.**7**	**0**.**7**	**20**.**3**

**Table 3:** QTL mapping results.

	Trait	LG	Peak (cM)	LOD	*R*^*2*^ (%)	a	1.5LOD Left	1.5LOD Right	Method
Salinity	S15	2	60	3.83	23	0.110	49	64	RQTL_Scanone
		6	52.686	4.97	10	0.077	50	52.7	MapQTL_MQM
		23	12.585	3.96	9.4	−0.048	7.6	12.6	MapQTL_MQM
	S20	1	54.123	5.81	11.2	−0.075	53	55	MapQTL_MQM
		6	63.964	4.62	9.6	0.068	62	64	MapQTL_MQM
		21	74.591	4.4	10.7	0.117	70.7	76	MapQTL_MQM
		24	71.624	4.89	10.2	−0.099	68.6	71.6	MapQTL_MQM
		26	36.69	3.38	6.8	−0.090	31.4	36.7	MapQTL_MQM
		26	56.356	3.2	6.4	0.090	51.4	56.3	MapQTL_MQM
	S20/S15	7	74.093	4.18	27	−0.033	68.4	86	MapQTL_MQM
	S34/S15	2	60.456	9.43	32.1	−0.029	60	64	MapQTL_MQM
		7	109.287	3.83	11	−0.007	108	113	MapQTL_MQM
		22	47.593	4.1	10.6	0.016	44	47.6	MapQTL_MQM
		22	58.867	3.53	9.7	−0.015	50	59	MapQTL_MQM
	S34/S20	1	13.812	3.95	5.8	−0.008	11.2	13.9	MapQTL_MQM
		1	54.123	15.83	33.6	0.020	54	56	MapQTL_MQM
		4	70.291	7.9	13.3	0.018	65	70.3	MapQTL_MQM
		4	83.254	8.62	15.2	−0.022	82.3	83.3	MapQTL_MQM
		11	30.17	7.61	12.7	0.015	28.8	30.2	MapQTL_MQM
		16	0	4.93	8	0.011	0	0	MapQTL_MQM
		16	21.773	6.43	10.4	−0.013	10.8	21.8	MapQTL_MQM
		26	9.778	5.63	8.5	−0.012	8.6	11.5	MapQTL_MQM
		26	56.356	6.61	10.5	−0.016	50.5	56.4	MapQTL_MQM
		26	80.621	6.39	9.6	0.012	74.4	80.7	MapQTL_MQM
	Survival_S15	23	17	3.75	5.6	−0.010	7	37.7	RQTL_Scanone
	Survival_S20	11	91.953	6.48	13	−10.408	91.9	92.3	MapQTL_MQM
		19	50.116	3.52	6.3	−7.211	45.5	50.4	MapQTL_MQM
		22	37.558	4.6	6.7	−13.560	36.5	41	MapQTL_MQM
Temperature	T26	9	32.541	4.18	19.5	0.188	31	34	MapQTL_MQM
	T28	2	57.891	9.54	21.3	0.058	57.5	57.9	MapQTL_MQM
		4	17.573	3.65	6.8	−0.040	11.3	18.6	MapQTL_MQM
		4	91.605	13.35	33.6	0.081	91.6	94.3	MapQTL_MQM
		14	0	5.8	11.4	−0.045	0	0	MapQTL_MQM
		18	15.042	4.83	9.3	0.037	15	15.1	MapQTL_MQM
		24	37.7	3.72	7	0.014	33	63	RQTL_Scanone
	T13/T26	5	99.023	3.65	13.4	−0.010	94	101	MapQTL_MQM
	T13/T28	26	111.259	3.26	12.7	0.012	90	112	MapQTL_MQM
	T26/T28	6	63.964	4.63	18.4	0.069	63.9	64.1	MapQTL_MQM
	Sex	13	40.1	52.1	75	0.500	39	41	RQTL_Scanone

T13, T26 and T28 correspond to growth rate phenotypes for temperatures 13 °C, 26 °C and 28 °C respectively. S34, S20 and S15 correspond to growth rate phenotypes for 34‰ salinity, 20‰ and 15‰ salinity respectively. Plasticity traits are represented by a letter followed by a number and a slash, then another letter and number. For example, S34/S20 corresponds to plasticity represented by slope of the reaction norm between the two conditions salinity 34‰ condition and salinity 20‰ condition. Surv_S15 and Surv_S20 correspond to survival trait for conditions salinity 15‰ and salinity 20‰.

In the method column, MapQTL_MQM means that the QTL was detected by both MapQTL and R/qtl, RQTL_Scanone means that the QTL was only detected by R/qtl.

a: estimated additive effect = (mu_A − mu_B)/2. Mu_A: the estimated mean of the distribution of the quantitative trait associated with the “a” genotype which is the genotype of the male parent. *R*^*2*^: the percentage of the variance explained for by the QTL. 1.5LOD_Left and 1.5LOD_Right represent left and right limits of the 1.5-LOD support interval.

**Table 4:** Pearson’s correlation coefficients between main studied traits.

	T13	T26	T28	S34	S20
T26	0.095				
T28	−0.017	0.003			
S34	0.038	0.217*	−0.05		
S20	−0.2	−0.26*	0.163	0.025	
S15	0.149	0.061	0.202	0.024	0.531****

See Table 2 for trait details.

*Significant at 0.05.

****Significance <0.0001.

**Table 5:** List of the top 20 GO terms identified by the test for GO term enrichment in genes located within the QTL intervals identified in this study.

GO ID	Term	Annotated	Significant	Expected	Rank in classic	p-value classic
GO:0015698	inorganic anion transport	3	2	0.15	1	0.007
GO:0006820	anion transport	4	2	0.2	2	0.014
GO:0015031	protein transport	22	4	1.1	3	0.02
GO:0008104	protein localization	24	4	1.2	5	0.027
GO:0006396	RNA processing	6	2	0.3	8	0.032
GO:0006886	intracellular protein transport	17	3	0.85	9	0.048
GO:0006605	protein targeting	8	2	0.4	12	0.056
GO:0072594	establishment of protein localization	8	2	0.4	13	0.056
GO:1902580	single-organism cellular localization	9	2	0.45	15	0.07
GO:0006811	ion transport	21	3	1.05	16	0.082
GO:0016482	cytoplasmic transport	11	2	0.55	17	0.1
GO:0006810	transport	103	8	5.14	22	0.128
GO:0051234	establishment of localization	104	8	5.19	23	0.133
GO:0009148	pyrimidine nucleoside triphosphate biosynthesis	3	1	0.15	25	0.142
GO:0043094	cellular metabolic compound salvage	3	1	0.15	26	0.142
GO:0007005	mitochondrion organization	3	1	0.15	24	0.142
GO:0044743	intracellular protein transmembrane importation	3	1	0.15	27	0.142
GO:0009147	pyrimidine nucleoside triphosphate metabolism	3	1	0.15	28	0.142
GO:0017038	protein import	3	1	0.15	29	0.142
GO:0051179	localization	106	8	5.29	32	0.144

See materials and methods for details.
