# Supplementary material for: High-density genetic map and identification of QTLs for responses to temperature and salinity stresses in the model brown alga Ectocarpus
Source: Sci Rep. 2017 Mar 3;7:43241. doi: 10.1038/srep43241 (PMC5335252; doi:10.1038/srep43241)
Supplement: Supplementary Information [file srep43241-s1.pdf]

High-density genetic map and identification of QTLs for responses to temperature and salinity stresses in the model brown alga *Ectocarpus*

Komlan Avia[1,2*], Susana M. Coelho[1], Gabriel J. Montecinos[1,2,3], Alexandre Cormier[1], Fiona Lerck[1], Stéphane Mauger[2], Sylvain Faugeron[2,3], Myriam Valero[2], J. Mark Cock[1] and Pierre Boudry[4]

[1]Algal Genetics Group, UMR 8227, CNRS, Sorbonne Universités, UPMC, Station Biologique Roscoff, CS 90074, 29688 Roscoff, France.
[2]UMI 3614 Evolutionary Biology and Ecology of Algae, CNRS, Sorbonne Universités, UPMC, Pontificia Universidad Católica de Chile, Universidad Austral de Chile, Station Biologique Roscoff, CS 90074, 29688 Roscoff, France.
[3]Centro de Conservación Marina and CeBiB, Facultad de Ciencias Biológicas, Pontificia Universidad Católica de Chile, Casilla 114-D, Santiago, Chile.
[4]Ifremer, Laboratoire des Sciences de l'Environnement Marin (UMR 6539 LEMAR, UBO, CNRS, IRD, Ifremer), Centre Bretagne – ZI de la Pointe du Diable, CS 10070, 29280 Plouzané, France.
[*]Corresponding author: komlan.avia@sb-roscoff.fr

Supplementary tables

LGs	pos 1f	pos 2f	lod.full	lod.fv1	lod.int	pos 1a	pos 2a	lod.add	lod.av1	Traits	QTLs
LG1:LG14	6	64	6.18	4.22	2.77	9	90	3.4	1.444	T13, T26, T28, T13/T28, T26/T28, Surv_S20, S15, S34/S15, S20/S15	No
LG4:LG9	0	0	5	3.09	0.923	0	0	4.08	2.168	T13, T13/T26, T13/T28, S34, S20/S15	No
LG4:LG12	0	112	4.4	2.49	0.113	0	112	4.29	2.375	T26, T13/T26, T13/T28, Surv_S15, S20, S20/S15	No
LG4:LG20	3	16	5.02	3.1	0.986	0	81	4.03	2.118	T13, T13/T26, Surv_S2, S20/S150	No
LG5:LG5	14	71	4.88	3.55	0.695	11	14	4.19	2.859	Surv_S20, T26/T28	No
LG5:LG14	115	96	5.58	3.62	1.59	115	91	3.99	2.032	T13, T13/T26, T13/T28, Surv_S15	No
LG5:LG20	117	20	4.89	3.56	1.5	84	81	3.39	2.059	T28, T26/T28, Surv_S20, Surv_S15	No
LG5:LG21	11	71	6.08	4.23	2.56	14	77	3.52	1.664	T13, T13/T26, T13/T28, S20	Yes
LG5:LG25	116	17	6.47	5.14	3.7	14	37	2.77	1.437	T13, T13/T28, Surv_S20	No
LG9:LG9	83	86	4.76	3.32	1.01	6	8	3.76	2.31	T13, T13/T26, S34, S15, S34/S20, S20/S15	No
LG9:LG27	57	44	6.37	4.18	3.07	0	54	3.29	1.102	T13, T13/T26, T13/T28, Surv_S20, S20/S15, S34/S15	No
LG11:LG11	39	79	5.07	3.04	0.742	75	78	4.32	2.301	T13, T13/T26, T13/T28, Surv_S20	No
LG12:LG12	58	59	9.03	7.67	3.38	57	59	5.66	4.29	T13, T13/T26, T13/T28, S20/S15	No
LG12:LG23	54	7	4.88	2.74	0.665	54	8	4.22	2.079	S34, S20, S20/S15	Yes
LG12:LG26	55	10	5.47	4.1	2.84	112	111	2.63	1.259	T26, T26/T28, Surv_S15	No
LG14:LG23	90	7	5.18	3.04	1.02	90	7	4.17	2.028	T13, T13/T26, T13/T28, Surv_S20	Yes
LG18:LG18	21	23	4.79	3.95	1.55	20	24	3.24	2.397	T13, T26, T28, T13/T28, T26/T28, S34, S34/S20, S20/S15	No
LG20:LG26	81	107	5.29	4.13	2.43	81	112	2.85	1.699	T13, T13/T26, T13/T28, S20, S15, Surv_S15, S20, S15	Yes

LG23:LG23	12	44	5.27	3.13	1.12	34	36	4.15	2.009	T13, T26, T13/T28, S15, S20/S15, T26/T28, S34, S34/S20	Yes
LG23:LG26	4	83	6.61	4.47	3.66	7	80	2.94	0.806	T13, T13/T28, S34/S15	No
LG11:LG19	88	50	5.19	4.03	3.3064	90	66	1.88	0.728	T13, T13/T26, T13/T28, S34, S15, S34/S15	Yes

Table S1. Epistatic effects between stress response loci.
pos1f: estimated position of the QTL on the first LG under the full model.
pos2f: estimated position of the QTL on the second LG under the full model.
lod.full: measures the improvement in the fit of the full two-locus model over the null model, and indicates evidence for at least one QTL, with allowance for interaction.
lod.fv1: measures the improvement comparing the full model with QTL on chromosomes j and k to the single-QTL model, with a single QTL on either chromosome j or k. Thus, it indicates evidence for a second QTL, allowing for the possibility of epistasis.
lod.int: measures the improvement comparing the full model with QTL on chromosomes j and k to the additive model with QTL on chromosomes j and k, and so indicates evidence for an interaction between QTL on chromosomes j and k, assuming that there is precisely one QTL on each chromosome (or, for j = k, that there are two QTL on the chromosome).
pos1a: estimated position of the QTL on the first LG under the additive model.
pos2a: estimated position of the QTL on the second LG under the additive model.
lod.add: measures the improvement in the fit of the two-locus additive model, and indicates evidence for at least one QTL, assuming no interaction.
lod.av1: measures the improvement comparing the additive model with QTL on chromosomes j and k to the single-QTL model, with a single QTL on either chromosome j or k. Thus, it indicates evidence for a second QTL, assuming no epistasis.
There was a total of 40 unique positions involved in the epistatic interactions (LG and position in cM linked by an "_" symbol, so LG_pos1f or LG_pos2f with no duplicates):
(LG1_6, LG11_39, LG11_79, LG11_88, LG12_112, LG12_54, LG12_55, LG12_58, LG12_59, LG14_64, LG14_90, LG14_96, LG18_21, LG18_23, LG19_50, LG20_16, LG20_20, LG20_81, LG21_71, LG23_12, LG23_4, LG23_44, LG23_7, LG25_17, LG26_10, LG26_107, LG26_83, LG27_44, LG4_0, LG4_3, LG5_11, LG5_115, LG5_116, LG5_117, LG5_14, LG5_71, LG9_0, LG9_57, LG9_83, LG9_86). But based on the linkage map, LG12_54 and LG12_55 correspond the same map position (there is a position at cM 54.615 and this is the case also for LG12_58 and LG12_59 (map position 58.368). Therefore, only one position was retained for these two cases, leading to 38 unique positions:

LG1_6, LG11_39, LG11_79, LG11_88, LG12_112, LG12_54, LG12_58, LG14_64, LG14_90, LG14_96, LG18_21, LG18_23, LG19_50, LG20_16, LG20_20, LG20_81, LG21_71, LG23_12, LG23_4, LG23_44, LG23_7, LG25_17, LG26_10, LG26_107, LG26_83, LG27_44, LG4_0, LG4_3, LG5_11, LG5_115, LG5_116, LG5_117, LG5_14, LG5_71, LG9_0, LG9_57, LG9_83, LG9_86.

Supplementary figures

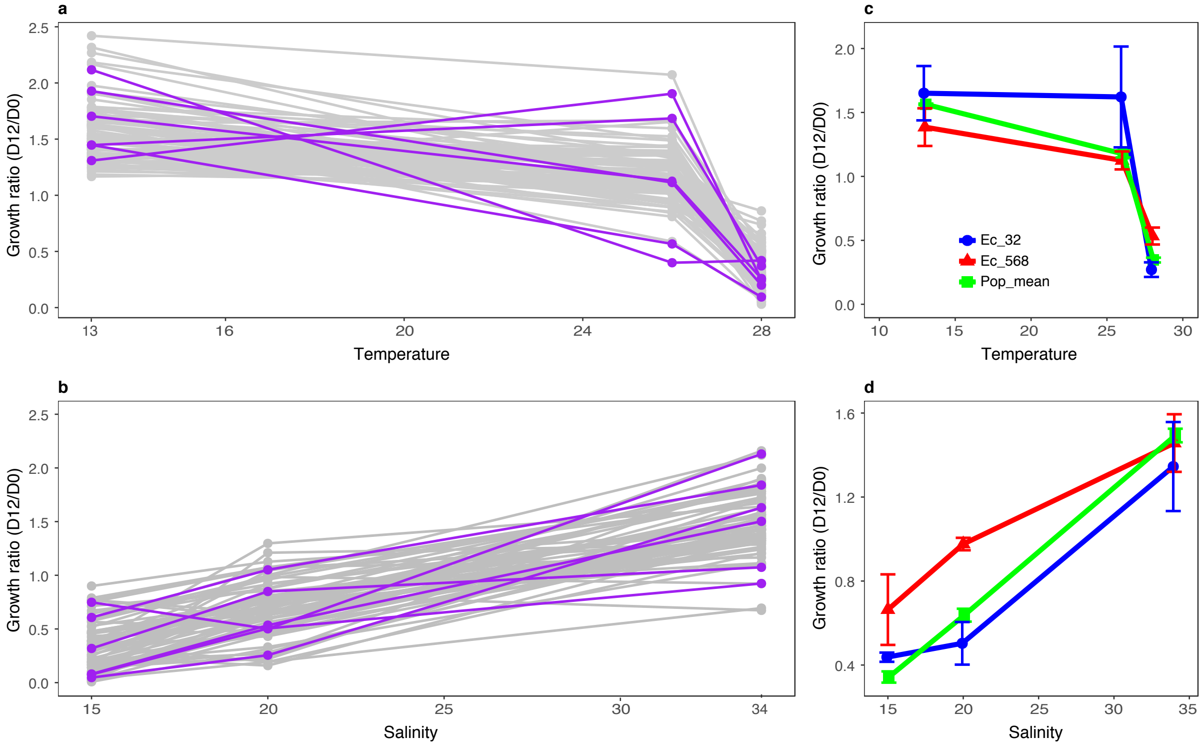

Figure S1. Reaction norms of the mapping family for temperature and salinity. Reaction norms of individual strains under the three temperature conditions (a) and the three salinity conditions (b). Some of the reactions norms are colored in purple to emphasise the fact that they cross. Panels c and d show the parental reaction norms (blue for the male parent and red for the female parent) along with the population mean reaction norm (in green) for temperature and salinity respectively. Error bars indicate standard errors.

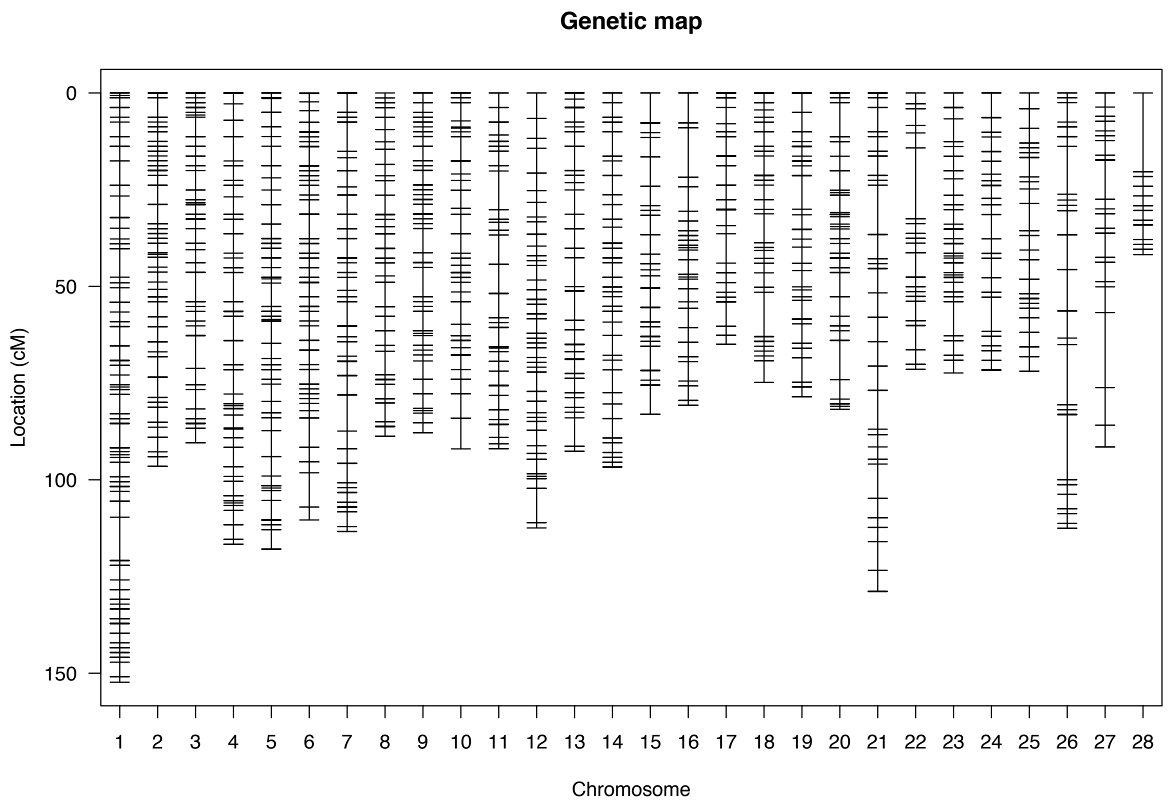

Figure S2. Representation of the ddRAD-seq-based genetic map of *Ectocarpus* sp. Marker positions are represented by dashes on each linkage group.

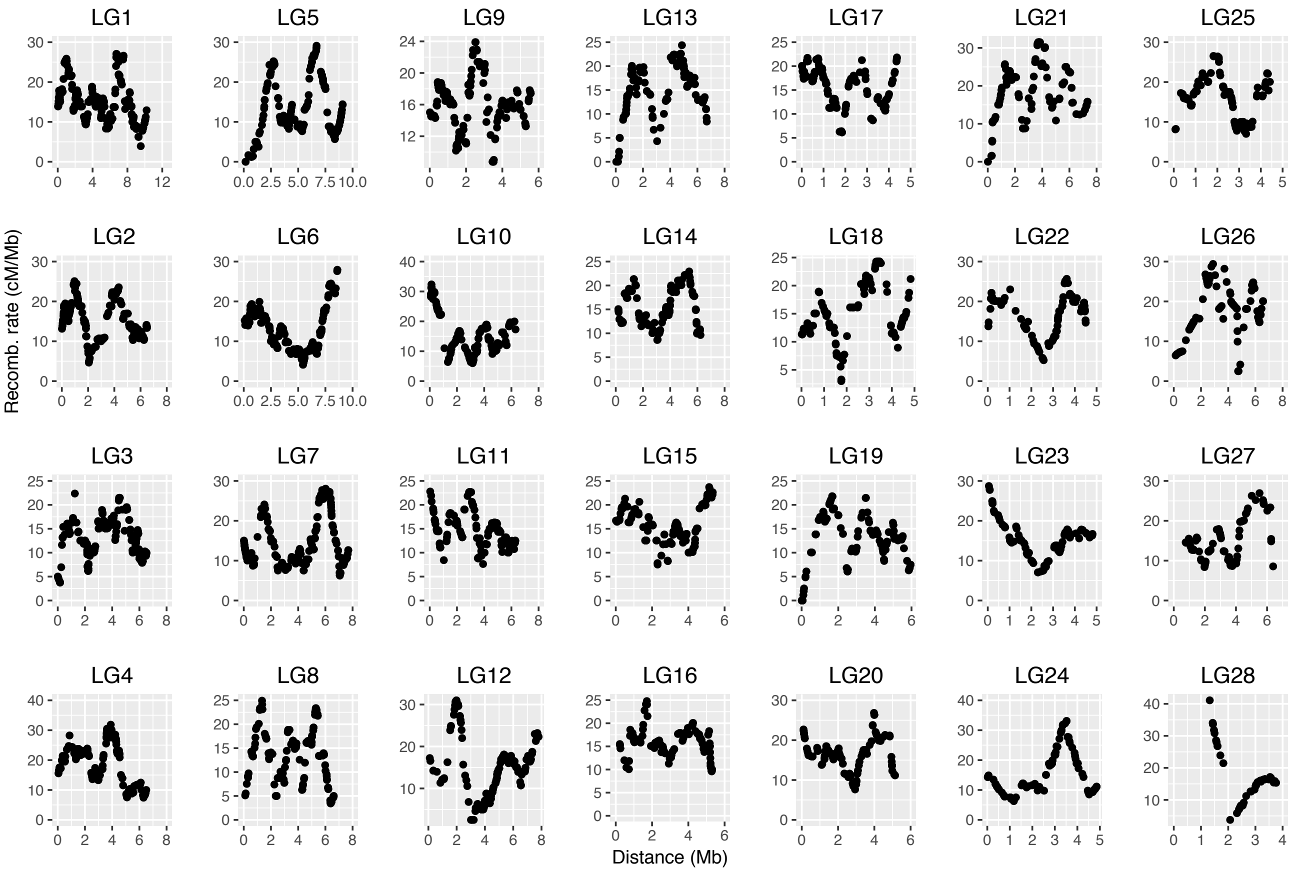

Figure S3. Average recombination rate along each linkage group in 1 Mbp sliding windows.

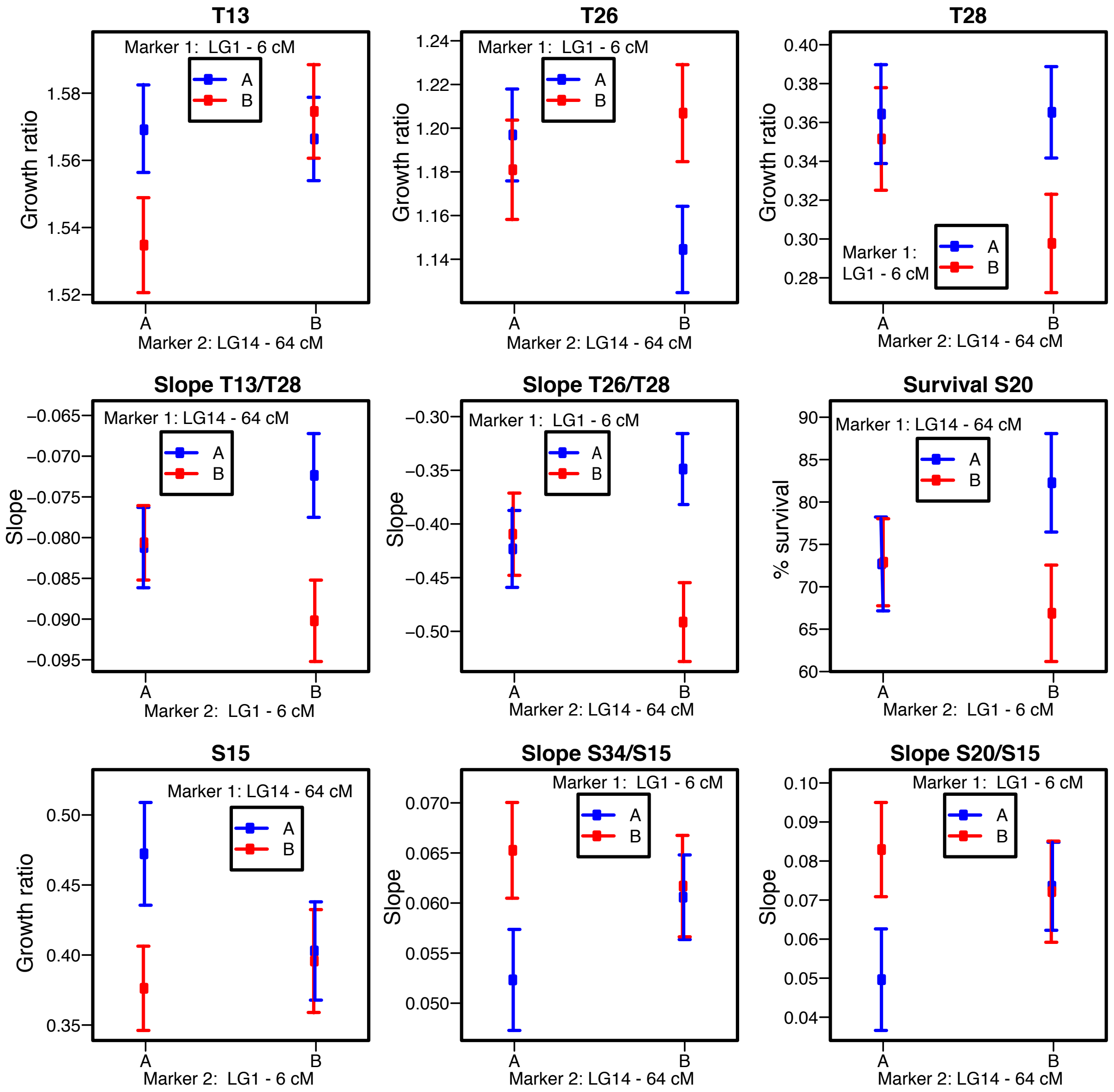

Figure S4. Examples of epistatic interactions.
Estimated growth ratio, slope of reaction norm or survival percentage as a function of genotypes of two interacting markers (one at 6 cM on LG1 and the other one at 64 cM on LG14). In each case, the marker 1 is represented in color (with blue color for the A genotype deriving from the male parent and red color for the B genotype deriving from the female parent as indicated in the interior legend). The squares represent the phenotypic mean values and error bars indicate the standard errors. The marker 2 is represented with its two genotypes on the x-axis.
As an example, in the case of trait T28, the result of epistasis between LG1 and LG14 showed that the locus at 6 cM on LG1 had an effect only in the presence of the B genotype (A and B genotypes derived from the male and female parents, respectively) of the locus at 64 cM on LG14. In the presence of the A genotype at the locus on LG14, no significant difference was observed between the growth ratio of individuals carrying the A genotype and those carrying the B genotype at the locus on LG1 (overlapping error bars).

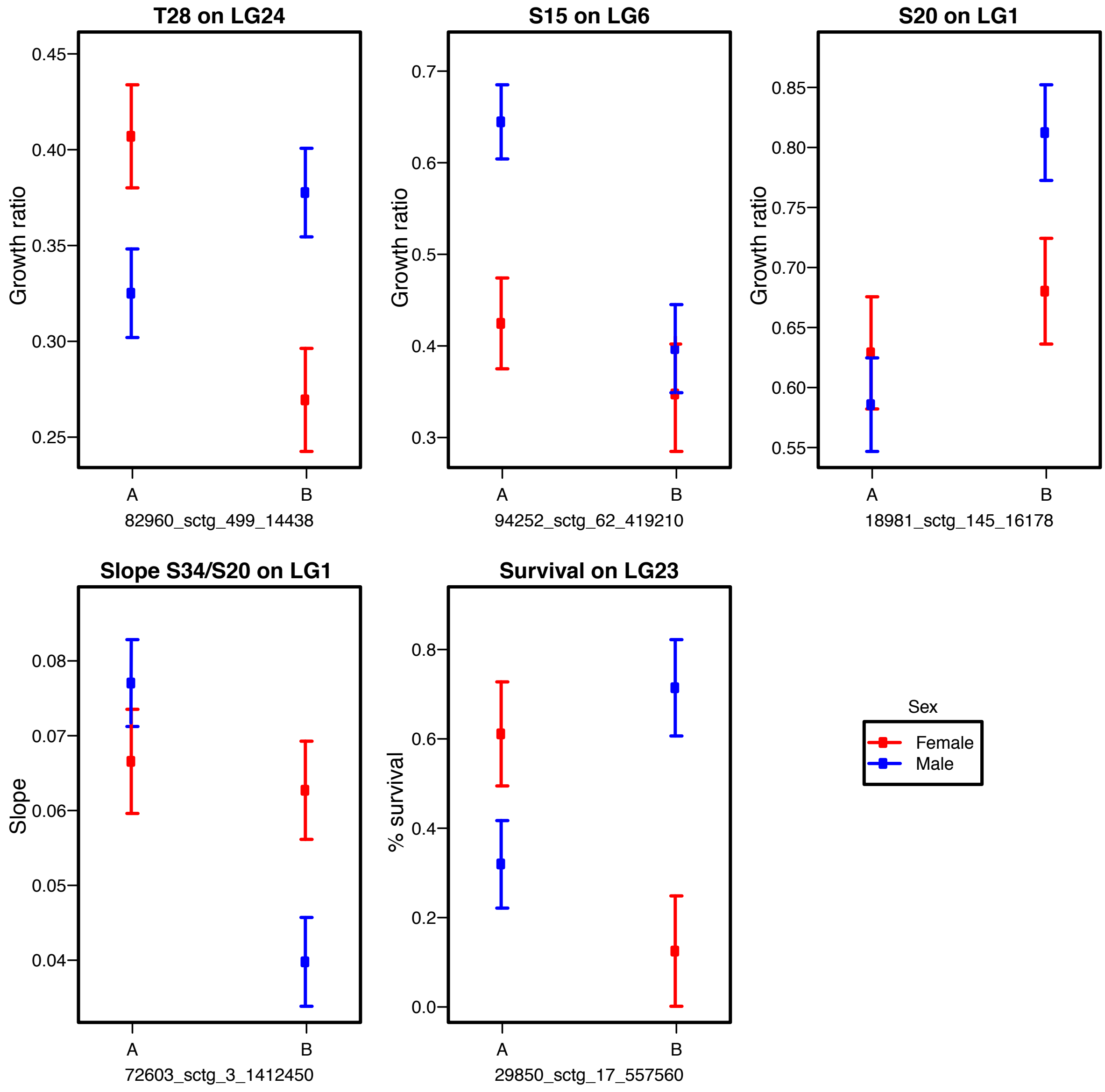

Figure S5. QTL x sex interactions.
Plots of the estimated population average growth ratio, slope of reaction norm or survival percentage as a function of sex (with males in blue and females in red). The x-axis indicates the two genotypes (A allele derived from the male parent and B allele derived from the female parent) of the nearest marker to the peak LOD score. The name of that nearest marker is shown under the x-axis (e.g. 82960_sctg_499_14438). The y-axis indicates the phenotype (growth rate, slope of the reaction norm or percent survival), corresponding to names above each graph. The squares indicate the phenotypic mean values and error bars indicate the standard errors.

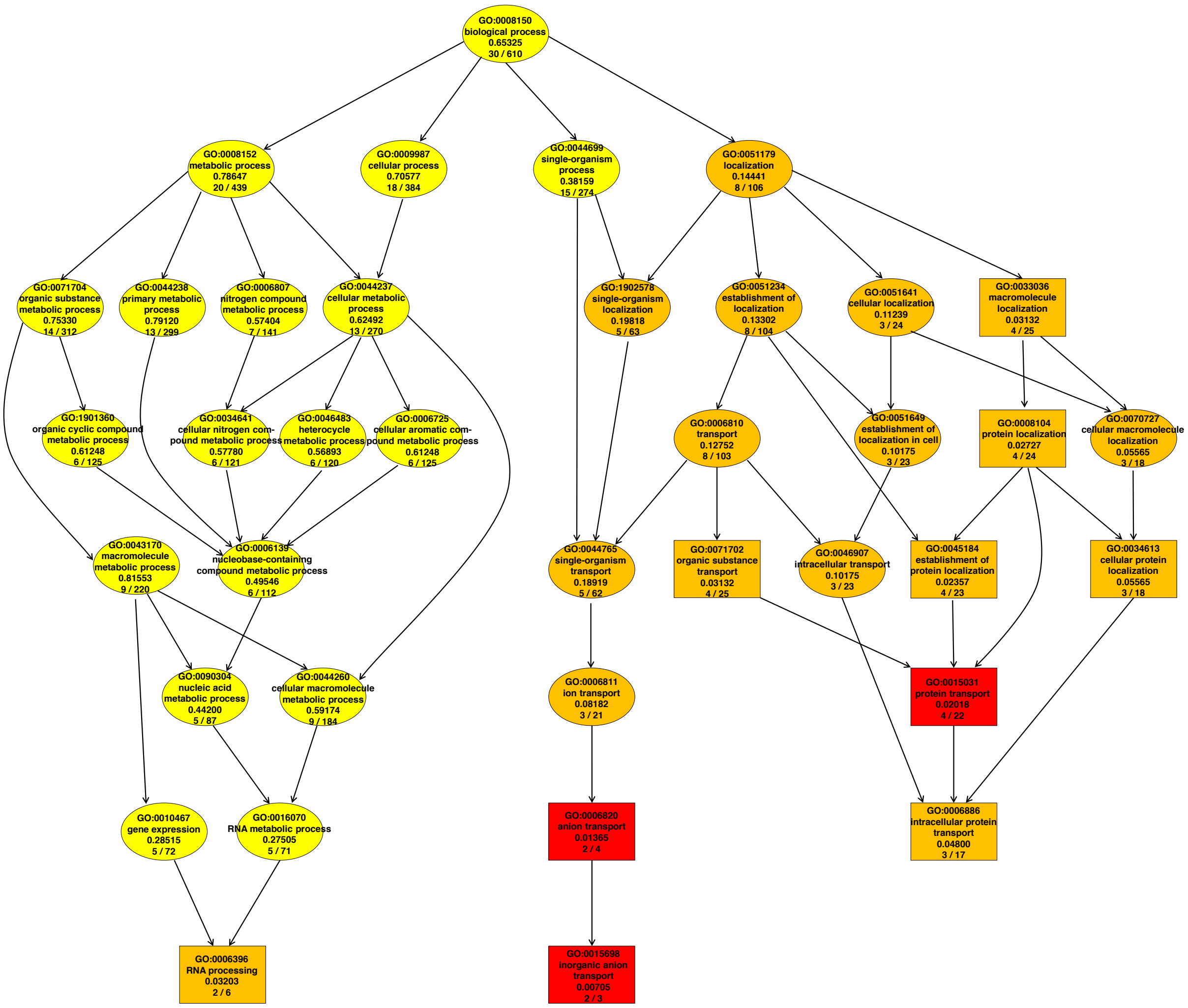

Figure S6. Subgraph induced by the top 10 GO terms identified by the “classic” algorithm for scoring GO terms enrichment test.
Significant nodes (10 nodes) are represented as rectangles and the rest of the nodes as circles. Colors represent relative significance, ranging from dark red (most significant) to bright yellow (least significant). For each node, some basic information is displayed: the GO identifier and name, the raw p-value and at the bottom, the number of significant genes / the total number of genes annotated to the respective GO term.
